# Evaluating the Impact of Massage Therapy on Performance and Well-Being in Taekwondo Practitioners: A Systematic Review

**DOI:** 10.3390/ijerph22050742

**Published:** 2025-05-08

**Authors:** Musa L. Mathunjwa, S’bongile Mahlangu, Monoem Haddad

**Affiliations:** 1 Department of Human Movement Science, University of Zululand, Private Bag X1001, KwaDlangezwa 3886, South Africa; mathunjwam@unizulu.ac.za (M.L.M.); sbongilemahlangu055@gmail.com (S.M.); 2 Sport Coaching Department, College of Sport Sciences, Qatar University, Doha P.O. Box 2713, Qatar

**Keywords:** manual therapy, combat sports, athletic recovery, injury mitigation, mental resilience

## Abstract

Background and objectives: Taekwondo is a physically intense martial art that demands strength, agility, and mental focus, often leading to physical and psychological strain. While massage therapy is recognized for enhancing muscle recovery, reducing injury risk, alleviating stress, and improving sleep quality in athletes, its specific effects on Taekwondo athletes remain underexplored. This review aims to evaluate the impact of massage therapy on physical recovery, injury prevention, and psychological well-being in Taekwondo athletes. Methods: A systematic literature search was conducted across Science Direct, Google Scholar, Sport Discus, and PubMed, using MeSH terms including “massage therapy”, “Taekwondo athletes”, “muscle recovery”, “injury prevention”, “psychological well-being”, “Swedish massage”, “deep tissue massage”, “sports massage”, “performance optimization”, and “sleep quality”. Results: Thirteen peer-reviewed studies met the inclusion criteria. The findings suggest that massage therapy improves muscle recovery, enhances flexibility and balance, supports injury prevention, and contributes positively to athletes’ psychological states, including mood and anxiety reduction. Conclusions: Massage therapy appears to be an effective intervention for enhancing both performance and well-being in Taekwondo athletes. Future studies should aim to standardize massage protocols and investigate long-term effects across training and competition periods.

## 1. Introduction

Taekwondo, a martial art originating in Korea, was initially developed to enhance combat abilities among soldiers and individuals [1]. Following the Korean War, it spread internationally and evolved into modern sport by the late 1950s [1]. Its inclusion in the Olympic Games in 1994 solidified its global prominence, making it one of the most widely practiced martial arts today [1]. Taekwondo is characterized by its dynamic, high-speed kicks and requires exceptional speed, power, and agility [2]. Competitors must combine physical prowess with strategic thinking, mental focus, and technical skill to succeed in bouts that alternate between intense bursts of action and periods of relative inactivity [2,3].

The demanding nature of Taekwondo necessitates rigorous physical preparation and mental resilience. Physical attributes such as aerobic and anaerobic power, muscular strength, flexibility, and agility are critical for success [2,4]. Psychological factors are equally important, as athletes must manage stress, maintain focus, and adapt to the high-pressure environment of competition [2,3]. These physical and mental demands make effective recovery essential for sustaining peak performance.

Massage therapy has gained recognition in sports medicine as a valuable tool for enhancing recovery and performance [5,6]. It is widely used in athletic populations to improve muscle recovery, prevent injuries, and alleviate stress, offering both physical and psychological benefits [7,8]. For Taekwondo athletes, who face unique challenges such as muscle fatigue from repeated high-intensity movements and mental strain from strategic decision-making, massage therapy could play a pivotal role. Research indicates that massage therapy promotes muscle relaxation, enhances circulation, and reduces delayed onset muscle soreness (DOMS), contributing to faster recovery after intense training or competition [9,10]. Additionally, the psychological benefits—such as reduced anxiety, improved mood, and heightened relaxation—are particularly relevant for athletes navigating the mental pressures of elite competition [8,9].

The physical and mental demands of Taekwondo make it essential for athletes to maintain optimal health and well-being to achieve peak performance [2,4,11,12]. With growing scientific support for complementary therapies in sports medicine, massage therapy has emerged as a promising tool for enhancing recovery and performance [13]. While there is existing literature supporting the benefits of massage therapy in various sports disciplines [14,15,16], there is a lack of comprehensive reviews specifically focusing on its impact on Taekwondo athletes.

This review hypothesizes that massage therapy has a positive impact on both physical performance and psychological well-being in Taekwondo athletes. The significance of this study lies in its potential to guide evidence-based recovery and performance strategies tailored to the specific demands of Taekwondo. By identifying and analyzing relevant studies, this review aims to offer practical insights for athletes, coaches, and practitioners, and to support the development of standardized massage protocols for this sport.

This review aims to fill this gap by providing a comprehensive assessment of the existing literature on the effects of massage therapy on Taekwondo athletes’ performance and well-being. By synthesizing the available evidence, this review aims to provide valuable insights and recommendations for athletes, coaches, and sports medicine professionals to optimize training regimens and improve competitive outcomes in Taekwondo.

## 2. Materials and Methods

### 2.1. Search Strategy

**Figure 1:**
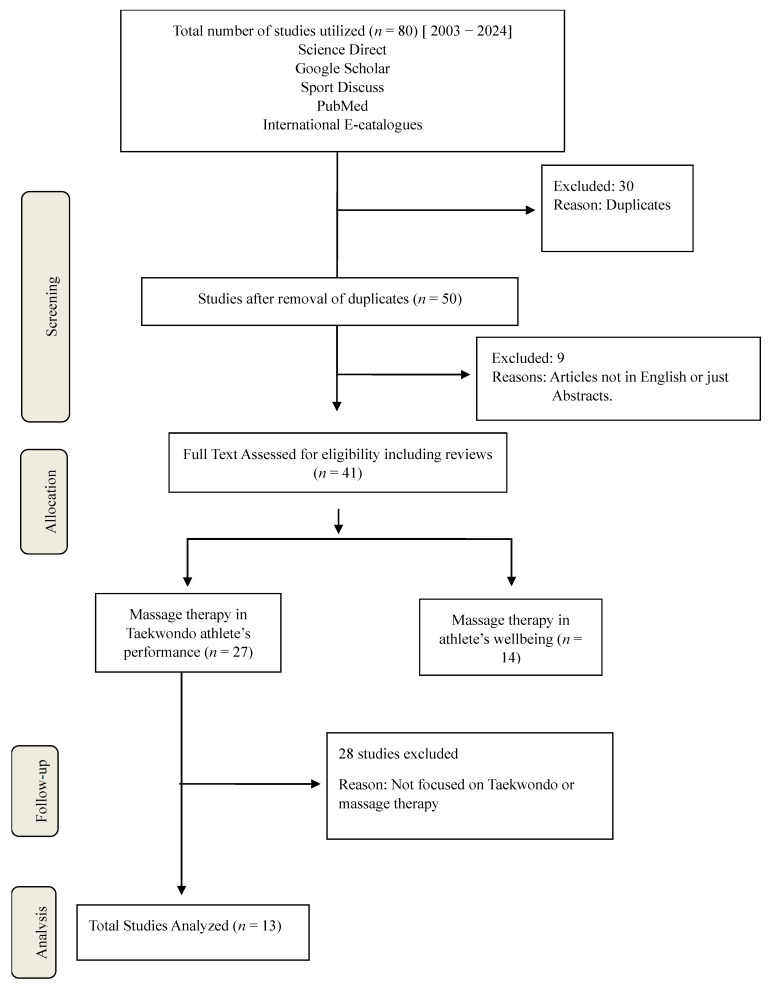
PRISMA Flow Chart of the study selection process.

A systematic literature search was conducted following the Preferred Reporting Items for Systematic Reviews and Meta-Analyses (PRISMA) guidelines. Studies were sourced from various databases, including Science Direct, Google Scholar, Sport Discuss, and PubMed. A comprehensive keyword search was performed using MeSH headings and keywords, including “massage therapy”, “Taekwondo athletes”, “muscle recovery”, “injury prevention”, “psychological well-being”, “Swedish massage”, “deep tissue massage”, “sports massage”, “performance optimization”, and “sleep quality”. The search was limited to studies published between 2003 and 2024. Only peer-reviewed articles written in English were considered, and the search results were categorized for discussion, as illustrated in Figure 1.

### 2.2. Inclusion and Exclusion Criteria

#### 2.2.1. Inclusion Criteria

Studies were included if they investigated massage interventions specifically applied to athletes, particularly Taekwondo practitioners. Eligible studies had to report on outcomes related to physical performance, muscle recovery, injury prevention, or psychological well-being. Interventions included various massage types such as Swedish massage, deep tissue massage, sports massage, pre-event massage, fascial therapy, and myofascial release. Studies were considered regardless of whether the massage was applied acutely (one session) or over a short-term period (multiple sessions). Session durations in the included studies generally ranged from 10 to 30 min, and studies with both male and female participants were accepted. Only peer-reviewed studies published in English between 2003 and 2024 were included.

#### 2.2.2. Exclusion Criteria

To maintain the relevance of the research included in this review, specific exclusion criteria were applied. Full-text articles in the English language were exclusively considered for this study. Additionally, articles were excluded if they were not focused on combat sports, including Taekwondo, or if they were non-peer-reviewed articles, conference papers, and reviews. Studies were also excluded if they did not provide evidence demonstrating the effects of massage therapy on the physical and mental well-being of Taekwondo athletes.

#### 2.2.3. Data Extraction

The studies that did not meet the inclusion criteria were excluded from the analysis. During full-text screening, articles were excluded for several reasons, including a lack of outcome relevance (e.g., not measuring performance or psychological effects), interventions not involving massage therapy, or studies focused on non-athlete populations. Additionally, non-peer-reviewed articles and conference abstracts were excluded. This rigorous filtering ensured that only studies meeting the defined inclusion criteria were analyzed.

Following the collection and analysis of significant data—which encompassed an examination of the effects of massage therapy on Taekwondo athletes’ performance and well-being the first author also evaluated eligibility for inclusion in the full-text article analysis. The final selection was approved by one of the co-authors, and any discrepancies were resolved through discussion until consensus was reached.

All the papers included in the analysis were sorted into two categories, either “massage therapy in Taekwondo” or “massage therapy in Taekwondo athletes’ well-being”, based on the respective journals or conferences of publication and associated keywords. The data-gathering process involved extracting information from the papers. To evaluate the contributions of each study to the effects of massage therapy, details about the analysis methodologies, types of massage therapy used, and outcomes related to physical and psychological well-being were collected. The findings were then systematically organized into distinct categories and placed within a comprehensive framework, which will serve as the structure for discussing the outcomes.

## 3. Results

This research utilized 80 full-text English-language papers obtained from 80 citations identified through electronic searches. After eliminating duplicates and reviewing full-text versions, 13 articles were retained for analysis. The study identified various types of massage and their effects on Taekwondo. Refer to Table 1 for the effects of massage on Taekwondo athletes’ performance and Table 2 for the effects of massage on athletes’ well-being.

While the primary focus of this review is on Taekwondo athletes, a small number of studies from different sports (e.g., wrestling, tennis, and rowing) were included to supplement the discussion of psychological outcomes. These were considered only when relevant Taekwondo-specific studies were unavailable and are clearly indicated in the table and discussion to maintain transparency.

## 4. Discussion

For centuries, massage has been used by healthcare professionals to help people with sickness and injuries [16]. It has both calming and invigorating characteristics that may impact an athlete’s performance at different stages like before, during, and after training or competition [10]. Massage, broadly described as the manipulation of soft tissue, can be utilized to promote recovery, prevent injuries, and serve as a passive warming technique before performances [10]. Massage therapy works through several physiological mechanisms that support both physical and mental recovery in athletes. It enhances local blood circulation and promotes lymphatic drainage, which helps remove metabolic waste products such as lactate and reduces inflammation and muscle soreness [5]. By manipulating soft tissues, massage also reduces muscle stiffness and tension, improving range of motion and flexibility [5]. On a neurological level, massage stimulates the parasympathetic nervous system, which lowers cortisol levels and induces a relaxation response, leading to reduced anxiety, improved mood, and better sleep quality [9]. There are different types of massage therapies including Swedish massage, sports massage, pre-event massage, myofascial release, fascial therapy, manual massage [26,27].

A study by Bayrakdaroğlu et al. [10] aimed to examine the effects of different durations of Swedish massage on the static and dynamic balance of Taekwondo athletes at different times of day. The study involved twelve Taekwondo athletes with more than five years of regular practice [10]. They underwent static and dynamic balance tests after different massage protocols, including a no-massage protocol (NMP), a five-minute massage protocol (5MMP), a ten-minute massage protocol (10MMP), and a fifteen-minute massage protocol (15MMP), administered twice a day (morning and evening) on non-consecutive days [10]. The findings revealed significant improvements in dynamic balance, particularly in the right foot, among Taekwondo athletes who received a 10MMP or 15MMP compared to those in the NMP group [10]. Interestingly, these improvements were observed regardless of the time of day when the massages were administered [10]. The results suggest that the duration of the massage plays a crucial role in enhancing dynamic balance in Taekwondo athletes, with longer massage durations leading to more pronounced improvements [10]. Furthermore, the study highlights the potential benefits of incorporating short-duration Swedish massages into pre-competition routines to optimize dynamic balance, a critical component of Taekwondo performance [10].

A study by Mohamed Shapie et al., aiming to compare the effects of static stretching and pre-event massage on kicking speed among Taekwondo athletes, found massage to be of particular importance in Taekwondo athletic performance [17]. Forty-five athletes aged 21 to 26 were divided into control, static stretching, and pre-event massage groups [17]. Kicking speed was measured before and after intervention using a 10 s kicking speed test [17]. The results showed that the pre-event massage group demonstrated a significant improvement in kicking speed compared to the control and static stretching groups [17]. These findings underscore the importance of massage therapy as a preparation method for Taekwondo athletes, offering potential benefits in enhancing performance. By manipulating fiber arrangement, promoting better blood flow, and aiding in the removal of biological wastes like lactic acid, pre-event massage appears to have a positive impact on athletic performance [17].

A study by Unalmis et al. investigated the impact of an eight-week fascial therapy program on the physical fitness parameters of Taekwondo athletes [3]. Aiming to enhance performance and recovery, this study enrolled 32 licensed taekwondo players who were actively engaged in training [3]. The participants were divided into two groups: a fascial therapy group (FTG) and a control group (CG) [3]. The study implemented a comprehensive fascial therapy program, consisting of osteopathic fascial therapy and relaxation techniques tailored specifically for the lower extremities of the athletes [3]. The therapy sessions, lasting approximately 30 min each, were conducted up to twice a week for eight weeks [3]. The participants in the FTG received the fascial interventions before their regular Taekwondo training sessions, while those in the CG did not undergo any additional intervention apart from their usual training regimen [3]. To assess the efficacy of the fascial therapy program, various physical fitness parameters were measured before and after the intervention period [3]. These parameters included flexibility, balance, speed (20m sprint), anaerobic power (assessed through vertical jump and standing long jump tests), among others [3]. Statistical analysis revealed significant improvements in these parameters among the participants in the FTG compared to the CG [3]. Specifically, the FTG exhibited significant enhancements in flexibility, balance, anaerobic power, and speed, as evidenced by lower sprint times and greater jump distances [3]. In contrast, the CG showed no significant changes in these parameters over the same period [3]. These findings underscore the potential benefits of incorporating structured fascial therapy programs into the training regimens of taekwondo athletes. By targeting the fascial system, these interventions may contribute to improved physical performance and overall athletic development, offering athletes a valuable tool for optimizing their competitive edge.

A study by Mehmet Akarsu et al., investigated the immediate effects of self-myofascial release using foam roller techniques and static stretching methods on the vertical jump performance of Taekwondo athletes [18]. Employing a cross-sectional design with a randomized controlled experimental approach, the study recruited 21 Taekwondo players who had undergone three years of training and volunteered to participate [18]. The study implemented protocols that targeted muscle groups such as the quadriceps, hamstrings, adductors, and gastrocnemius, for both self-myofascial release and static stretching exercises [18]. Notably, the results showed a significant improvement in vertical jump performance following self-myofascial release using foam roller exercises compared to both 5 min of slow-paced jogging and static stretching (*p* < 0.05) [18]. This indicates a promising immediate enhancement of Taekwondo athletes’ vertical jump performance through self-myofascial release, highlighting its potential significance in optimizing athletic abilities.

Another study on manual massage focused on its effects on the peak torque of the knee extensors after short-term intense continuous concentric–eccentric isokinetic exercise [19]. The study aimed to investigate the possible effects of manual massage on the concentric and eccentric peak torque of knee extensors when applied during intervals of exercise [19]. Twelve elite female Taekwondo athletes participated in the study [19]. Participants were subjected to continuous concentric–eccentric isokinetic exercise with manual massage applied to one extremity during breaks, while the other limb served as the control with passive intervals [19]. Peak torque was measured before and after the exercise protocol [19]. Results showed that for both groups, peak torque (concentric and eccentric) was reduced after the exercise protocol [19]. However, the massage group exhibited a significantly lower reduction for the eccentric type of muscle work (*p* < 0.05) [19]. Specifically, no statistical differences were noted in peak torque between extremities during the first two visits [20]. The findings indicated a significant difference in eccentric performance between limbs (F (1.22) = 8.27, *p* < 0.05) [19]. These results suggest that manual massage during isokinetic exercise intervals has an enhancing effect on the peak eccentric torque of the knee extensors, indicating its potential benefits in athletic performance and recovery [19].

A similar study on manual massage which investigated elite male athletes found that the application of manual massage during intervals of isokinetic exercise did not significantly improve the reduction in peak torque after exercise-induced muscle damage in the knee extensors [20]. Fourteen athletes participated in the study, undergoing continuous concentric and eccentric isokinetic exercises with manual massage applied to one leg during breaks, while the other leg served as a control with a passive interval [20]. Peak torque measurements were taken before and after the exercise protocol [20]. The results showed that both legs experienced a reduction in peak torque after the exercise, with the reduction being smaller in the leg receiving the massage [20]. However, this difference was not statistically significant (*p* > 0.05) [20]. Therefore, the application of manual massage during isokinetic exercise intervals did not improve the reduction in peak torque after exercise-induced muscle damage [20].

A study by Seo et al. [21] investigated the effects of electrical stimulation and massage therapy on blood lactate levels following anaerobic muscle fatigue in Taekwondo athletes. Conducted as a double-blind randomized controlled trial with 24 male collegiate athletes, the study aimed to compare the effectiveness of these interventions against a control group that rested post-exercise [21]. They found that massage significantly reduced blood lactate levels compared to those of the control group, indicating its efficacy in accelerating muscle recovery [21]. These findings suggest that massage therapy is effective in enhancing recovery, supporting its integration into post-training and post-competition routines for Taekwondo athletes to improve their performance and well-being [21]. In practical terms, these findings support the use of massage therapy as a viable and effective method to aid recovery in Taekwondo athletes, helping to clear lactic acid more efficiently than passive rest. This can lead to improved performance, reduced muscle soreness, and quicker readiness for subsequent training or competition.

Many coaches and athletes widely apply massage in sports events because they believe, based on their observations and experiences, that it offers several benefits for the body. These include enhanced blood circulation, decreased muscle tension and neurological excitability, and an improved overall sense of well-being. In the analysis of psychological well-being outcomes, insights were drawn from athletes in other sports disciplines due to a scarcity of Taekwondo-specific data in this area. These studies, while not directly related to Taekwondo, offer valuable comparative perspectives on the mental health effects of massage therapy.

A study by Zadkhosh et al. [22] that researched the effects of massage therapy on depression, anxiety, and stress in youth wrestlers found that after employing a 10-session intervention of 25 min sports massages, significant reductions in anxiety and stress were observed in the experimental group compared to the control group, both with *p* < 0.001. Using the DASS Inventory, the study measured these outcomes before and after the intervention [22]. However, depression scores showed only a slight decrease and were not statistically significant (*p* = 0.955) [22]. These results suggest that massage therapy may effectively reduce anxiety and stress levels among youth wrestlers, highlighting its potential benefits for mental health in athletic populations.

A study by Pa et al. [23] investigated how sports massage therapy impacts cortisol levels and anxiety before competitions among elite tennis athletes in Malaysia. The study used a controlled experimental design with 14 elite Malaysian tennis athletes [23]. The study employed a treatment group that received sports massage therapy and a control group that did not receive any massage [23]. Using saliva samples and standardized psychological questionnaires, the study found that sports massage significantly reduced cortisol levels and decreased pre-competition anxiety among athletes [23]. These results indicate that sports massage therapy effectively lowers the physiological stress response and improves mental readiness.

Aeini [24] investigated how massage therapy impacts fatigue and mood among female rowers. The primary focus of the study was to determine whether regular massage sessions can reduce fatigue and improve mood in female rowers [24]. Female rowers underwent 12 massage sessions, and standardized questionnaires were used to assess their fatigue levels and mood states before and after the intervention [24]. The results showed a significant decrease in fatigue levels among the female rowers who participated in the massage sessions [24]. This suggests that massage therapy is effective in alleviating physical fatigue. The study also observed a notable improvement in mood among the participants [24]. The mood assessments indicated reduced negative mood states such as tension and confusion and an overall increase in positive mood [24].

Parsakia et al. [25] investigated the impact of strength-based therapy on self-efficacy and life satisfaction among competitive athletes. The primary focus of the study was to determine whether an 8-week strength-based intervention could enhance psychological well-being by leveraging athletes’ personal strengths [25]. Fifty athletes aged 18–35 participated in the quasi-experimental study, which included weekly sessions focused on identifying personal strengths, setting goals, and building resilience [25]. Self-efficacy and life satisfaction were assessed using the General Self-Efficacy Scale and the Satisfaction with Life Scale at three intervals: pre-test, post-test, and one-month follow-up [25]. The results showed a significant increase in self-efficacy among participants who received the strength-based intervention compared to the control group [25]. This suggests that strength-based therapy can effectively boost an athlete’s belief in their ability to succeed. The study also found marked improvements in life satisfaction among the experimental group [25]. Athletes reported feeling more positive about their lives, with increased motivation and emotional resilience, as indicated by the follow-up data [25]. These findings highlight the potential of strength-based therapy as a valuable psychological tool in athletic settings [25].

Despite the overall positive trends, inconsistencies were observed across studies. For example, Sykaras et al. [20] found no statistically significant effect of manual massage on eccentric torque in male athletes, contrasting with significant improvements found in female athletes in a related study. Variability in intervention protocols, sample characteristics, and outcome measures likely contributed to these discrepancies. Such differences underscore the need for standardized massage protocols in future research.

These findings support the initial hypothesis that massage therapy positively affects both physical and psychological aspects of performance in Taekwondo athletes. The findings of this review suggest that massage therapy can be a valuable tool for enhancing both physical performance and psychological well-being among Taekwondo athletes. Coaches and support staff should consider integrating evidence-based massage techniques—such as Swedish massage, pre-event massage, or myofascial release—into training and recovery routines. Given that some protocols demonstrated benefits in session durations as short as 10 to 15 min, massage can feasibly be incorporated even in time-constrained settings. However, professional guidance is recommended to ensure proper technique, especially for interventions targeting deep tissue or fascial systems.

## 5. Limitations

This review has a few limitations that should be noted. Firstly, only twelve studies met the inclusion criteria, which means that the overall findings are based on a small number of sources. Most of the included studies also had small sample sizes, which may limit how well the results can be applied to the wider population of Taekwondo athletes.

Second, there were differences between the studies in terms of the type of massage used, how often the massage was applied, how long the sessions lasted, and the ways in which the results were measured. These differences made it difficult to compare the studies directly and to determine which massage methods are the most effective.

Third, although this review mainly focused on Taekwondo athletes, some studies involved athletes from other sports such as wrestling, tennis, and rowing. These were included for comparison, but their findings may not fully apply to Taekwondo.

In addition, most studies only looked at the short-term effects of massage. There is a need for future studies to explore the long-term effects over a full training season or competition period.

A further limitation is the absence of a formal risk of bias or quality grading using tools such as ROBIS or GRADE. Although only peer-reviewed and methodologically relevant studies were included, this limits the ability to assess internal validity and the strength of evidence across studies. Future reviews should incorporate structured quality appraisal tools to enhance methodological rigor.

## 6. Conclusions

This comprehensive review underscores the significant benefits of massage therapy for Taekwondo athletes, highlighting improvements in both physical performance and psychological well-being. The evidence from peer-reviewed studies reveals that various forms of massage therapy such as Swedish massage, pre-event massage, fascial therapy, myofascial release, and manual massage positively influence recovery, injury prevention, flexibility, balance, and psychological factors like anxiety and mood. For Taekwondo athletes and coaches, integrating massage therapy into training routines can optimize performance, reduce stress, and accelerate recovery. Future research should focus on refining these techniques and establishing evidence-based protocols tailored to the unique demands of Taekwondo. Furthermore, future research should aim to develop and evaluate standardized massage therapy protocols tailored to Taekwondo athletes, ensuring consistency in application and outcome assessment.

## Figures and Tables

**Table 1:** Effect of massage therapy on Taekwondo athletes’ performance.

Type of Massage	Study	Participants	Key Findings	Citation
**Swedish massage**	Bayrakdaroğlu et al.	12 Taekwondo athletes with more than five years of practice	Significant improvements in dynamic balance (right foot) were observed with 10 and 15 min protocols, regardless of the time of day (*p* < 0.05).	[10]
**Pre-event massage**	Mohamed Shapie et al.	45 athletes aged 21 to 26	Significant improvement in kicking speed was noted compared to the control and static stretching groups (*p* < 0.01).	[17]
**Fascial therapy**	Unalmis et al.	32 licensed Taekwondo players	Significant improvements in flexibility, balance, anaerobic power, and speed were found compared to the control group (*p* < 0.05).	[3]
**Myofascial release**	Mehmet Akarsu et al.	21 Taekwondo players	Significant improvement in vertical jump performance was observed after self-myofascial release compared to jogging and static stretching (*p* < 0.01).	[18]
**Manual massage**	Sykaras et al.	12 elite female Taekwondo athletes	Lower reduction in peak eccentric torque of knee extensors was noted compared to control (*p* < 0.05).	[19]
**Manual massage**	Sykaras	14 elite male athletes	No significant improvement in peak torque reduction after exercise-induced muscle damage was observed (*p* > 0.05).	[20]
**General Massage therapy**	Seo et al.	24 male collegiate athletes	Significant reduction in blood lactate levels post-exercise was observed compared to the control group (*p* < 0.01).	[21]

**Table 2:** Effect of massage therapy on Taekwondo athletes’ well-being.

Study	Participants	Key Findings	Citation
**Zadkhosh et al.**	Youth wrestlers	Significant reductions in anxiety and stress were observed after a 10-session sports massage intervention (*p* < 0.05).	[22]
**Pa et al.**	14 elite Malaysian tennis athletes	Significant reduction in cortisol levels and decreased pre-competition anxiety with sports massage therapy (*p* < 0.01).	[23]
**Aeini**	Female rowers	A significant decrease in fatigue and improvement in mood were observed after 12 massage sessions (*p* < 0.05).	[24]
**Parsakia et al.**	Athletes	Significant improvements in self-efficacy and life satisfaction were observed in the experimental group compared to the control group (*p* < 0.05)	[25]

## Data Availability

No new data were created.

## Abbreviations

The following abbreviations are used in this manuscript:

CG	Control group.
FTG	Fascial therapy group.
MMP	Minute massage protocol.
NMP	No Massage Protocol.
